# Chitosan-Based Nanoencapsulated Essential Oils: Potential Leads against Breast Cancer Cells in Preclinical Studies

**DOI:** 10.3390/polym16040478

**Published:** 2024-02-08

**Authors:** Wen-Nee Tan, Benedict Anak Samling, Woei-Yenn Tong, Nelson Jeng-Yeou Chear, Siti R. Yusof, Jun-Wei Lim, Joseph Tchamgoue, Chean-Ring Leong, Surash Ramanathan

**Affiliations:** 1 Chemistry Section, School of Distance Education, Universiti Sains Malaysia, Minden 11800, Penang, Malaysia; benedictsamling@gmail.com; 2 Faculty of Resource Science and Technology, Universiti Malaysia Sarawak, Kota Samarahan 94300, Sarawak, Malaysia; 3 Institute of Medical Science Technology, Universiti Kuala Lumpur, Kajang 43000, Selangor, Malaysia; 4 Centre for Drug Research, Universiti Sains Malaysia, Minden 11800, Penang, Malaysia; nelsonchear@usm.my (N.J.-Y.C.); sryusof@usm.my (S.R.Y.); srama@usm.my (S.R.); 5 HICoE-Centre for Biofuel and Biochemical Research, Institute of Self-Sustainable Building, Department of Fundamental and Applied Sciences, Universiti Teknologi PETRONAS, Seri Iskandar 32610, Perak Darul Ridzuan, Malaysia; junwei.lim@utp.edu.my; 6 Department of Biotechnology, Saveetha School of Engineering, Saveetha Institute of Medical and Technical Sciences, Saveetha University, Chennai 602105, India; 7 Department of Organic Chemistry, Faculty of Science, University of Yaoundé I, Yaoundé P.O. Box 812, Cameroon; joseph.tchamgoue@facsciences-uy1.cm; 8 Branch Campus Malaysian Institute of Chemical and Bioengineering Technology, Universiti Kuala Lumpur, Alor Gajah 78000, Melaka, Malaysia; crleong@unikl.edu.my

**Keywords:** breast cancer, chitosan, drug delivery, nanoencapsulated essential oils, therapeutics

## Abstract

Since ancient times, essential oils (EOs) derived from aromatic plants have played a significant role in promoting human health. EOs are widely used in biomedical applications due to their medicinal properties. EOs and their constituents have been extensively studied for treating various health-related disorders, including cancer. Nonetheless, their biomedical applications are limited due to several drawbacks. Recent advances in nanotechnology offer the potential for utilising EO-loaded nanoparticles in the treatment of various diseases. In this aspect, chitosan (CS) appears as an exceptional encapsulating agent owing to its beneficial attributes. This review highlights the use of bioactive EOs and their constituents against breast cancer cells. Challenges associated with the use of EOs in biomedical applications are addressed. Essential information on the benefits of CS as an encapsulant, the advantages of nanoencapsulated EOs, and the cytotoxic actions of CS-based nanoencapsulated EOs against breast cancer cells is emphasised. Overall, the nanodelivery of bioactive EOs employing polymeric CS represents a promising avenue against breast cancer cells in preclinical studies.

## 1. Introduction

Cancer is one of the leading causes of mortality worldwide, accounting for almost ten million deaths in 2020 [1]. Among cancer diseases, breast cancer is the most frequently diagnosed cancer. Breast cancer is a heterogeneous disease generally linked to oestrogen hormones. In addition, it was reported that 5–10% of breast cancer cases are linked to gene alterations. Breast cancer is more common in women between the ages of 65 and 80. However, invasive breast cancer incidence is seen in women under the age of 50. According to studies, early-stage breast cancer can be cured in about 70% of patients, but metastatic breast cancer is typically incurable [2]. Currently, the main treatments for breast cancer include chemotherapy, radiation therapy, and surgery. However, the adverse side effects of conventional therapies have driven researchers to search for alternatives [3].

Regarding the important role of natural products as a source of biologically active constituents, essential oils (EOs) appear as potential candidates for combating human diseases. The use of plant EOs has been evidenced for thousands of years. It was recorded that the Egyptians employed plant EOs for medicinal purposes as early as 4500 BC. Since then, EOs have been produced commercially owing to their widespread use and medicinal value [4,5]. Generally, EOs are a complex mixture of volatile aromatic constituents that are extracted from various plant parts. Due to their wide range of biological effects, EOs are attracting immense scientific attention and may be essential for the treatment of cancer [6,7]. However, EOs present some disadvantages in biomedical applications owing to their volatility, instability, and water insolubility and the heterogeneity in their chemical composition and biological effects [8,9].

In this context, encapsulation may overcome the shortcomings in preserving the bioactive constituents of EOs. Encapsulation can improve the stability, bioavailability, functional properties, and controlled release of bioactive constituents of EOs [8,10]. Additionally, adjusting the pH during encapsulation may enhance the stability of EOs. Encapsulation using biopolymers as carrier agents has attracted immense attention owing to their beneficial attributes. Among the wide range of biopolymers, chitosan (CS) has been widely employed in pharmaceutical settings, including for drug delivery. Generally, CS is the deacetylated form of chitin and is a generally recognized as safe (GRAS) material. It exhibits good properties such as biodegradability, bioavailability, biocompatibility, and non-toxicity [11,12]. Therefore, CS-based nanoencapsulated EOs offer an alternative approach to enhance the physical stability and bioavailability of bioactive EOs. The nanoscale particle size of EOs embedded in CS increased the surface-to-volume ratio, water solubility, and colloidal stability and resulted in a better controlled release of bioactive constituents [13,14]. In this review, we highlight the bioactive attributes of EOs and their constituents against breast cancer cells. The challenges associated with the biomedical applications of EOs are discussed. In addition, we emphasise the benefits of nanoencapsulated EOs, with a specific focus on CS-based nanoencapsulated EOs against breast cancer cells.

## 2. Essential Oils (EOs)

EOs are volatile organic constituents with low molecular weights extracted from leaves, buds, flowers, seeds, stems, fruits, roots, rhizomes, barks, and tubers [15,16,17,18]. Generally, EOs are insoluble in water but soluble in organic solvents [19,20]. Conventionally, EOs are extracted using hydrodistillation, steam distillation, or solvent extraction. Hydrodistillation is regarded as the simplest extraction technique. Plant materials are boiled with distilled water and connected to a Clevenger-type apparatus. Steam distillation, on the other hand, is conducted by passing steam through the plant materials. Hot vapours are then condensed to collect EOs. Solvent extraction involves the usage of organic solvent to macerate the plant materials, followed by filtration and solvent concentration [21,22]. Meanwhile, modern extraction techniques such as supercritical fluid extraction, microwave-assisted extraction, and ultrasonic-assisted extraction have improved the quality and yield of EOs. Shorter extraction time, lower energy consumption, and lesser solvent usage are among the notorious advantages of non-conventional extraction techniques for EOs [23,24]. Supercritical fluid extraction uses carbon dioxide as the solvent to pass through the plant materials. The collected EOs and the extracts are then separated by decompression [25]. Microwave-assisted extraction uses microwaves as the source of energy. It is environmentally friendly and requires a short extraction time [26]. Ultrasonic-assisted hydrodistillation involves the penetration across the plant cells via cavitation. This method improves the extraction efficiency and prevents the degradation of plant materials [27]. Extraction of plant EOs has been the focal point of research interest as it plays a key role in determining the type, amount, and chemical structures of the EO constituents [22].

For decades, EOs have been recognised as source of pharmaceutical agents. They possess a broad spectrum of biological activities, notably antioxidant, anti-inflammatory, anticancer, antibacterial, antiviral, antifungal, antimutagenic, antiparasitic, antimycotic, and antidiabetic activities [15,28,29]. Typically, these biological activities are mainly attributed to the predominance of major constituents. Nonetheless, previous studies have reported that interactions between different EO constituents may lead to additive or synergistic effects. An additive effect is defined as the sum of the individual effects of two or more constituents together. Meanwhile, a synergistic effect is defined as the combined effect of two or more constituents being greater than the sum of all of their individual effects [21]. Thus far, more than 3000 plant EOs have been extracted owing to their attractive biological activities. Nevertheless, the quality and yield of EOs are affected by factors such as plant parts, extraction methods, harvesting seasons, geographical locations, and postharvest storage conditions [30]. In industrial applications, EOs are widely used as flavouring agents in food and beverage, cosmetics, fragrances, and oral products [31]. For example, EOs extracted from citrus and lavender are commonly used in fragrances [32]. In soft drink manufacturing, EOs of cola, cinnamon, and vanilla are often employed [33]. Meanwhile, peppermint EOs are commonly used as the main ingredient in the manufacturing of oral products, such as mouthwash and toothpaste, confectionery, analgesic balms, chewing gums, and tobacco [34].

### 2.1. Constituents of EOs

EOs comprise more than 300 volatile organic constituents with molecular weights less than *m*/*z* 300 [5]. Generally, EOs consist of terpenes and phenylpropanoids [24]. Terpenes and terpenoids have been extensively investigated for their important roles in human health owing to their excellent therapeutic properties [35]. Terpenes are grouped according to the number of isoprene units in their structure. The isoprene units undergo head-to-tail condensation or rearrangement to give an array of terpenes. They are further classified into hemiterpenes (C5), monoterpenes (C10), sesquiterpenes (C15), diterpenes (C20), sesterterpenes (C25), triterpenes (C30), sesquarterpenes (C35), and tetraterpenes (C40). Terpenes are regarded as one of the most prominent groups of plant secondary metabolites. On the contrary, terpenoids are oxygen-containing terpenes that are synthesised via biochemical modifications and reactions [36]. They are categorised into alcohols, aldehydes, epoxides, esters, ether, ketones, and phenols [35].

**Figure 1:**
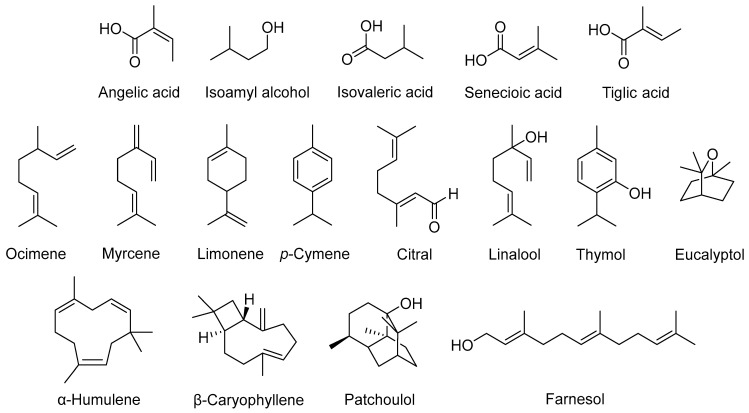
Chemical structures of common EO constituents.

Among terpenes, hemiterpenes are the simplest. They are regarded as a minor group in plant EOs, with a molecular formula of C_5_H_10_. Hemiterpenes are commonly released from conifers, oaks, and poplars. Some common hemiterpenes found in EOs are angelic acid, isoamyl alcohol, isovaleric acid, senecioic acid, and tiglic acid [35]. Monoterpenes comprise two units of isoprene and have the molecular formula of C_10_H_16_. They make up approximately 90% of total EO constituents [37]. Monoterpenes primarily control the release of specific odours from plants and are divided into acyclic and cyclic forms. For instance, ocimene and myrcene are acyclic monoterpenes, while limonene and p-cymene are cyclic monoterpenes [38]. Conversely, citral and linalool are common acyclic monoterpenoids, while cyclic monoterpenoids are represented by thymol and eucalyptol [36]. Sesquiterpenes, on the other hand, are less volatile than monoterpenes. Sesquiterpenes are derived from three isoprene units and have the molecular formula of C_15_H_24_. Similar to other terpenes, sesquiterpenes exist in both acyclic and cyclic forms. Sesquiterpenes and their derivatives are commonly detected in plant EOs. They are receiving much interest owing to their distinctive odour and flavour attributes. Examples of common sesquiterpenes and sesquiterpenoids are α-humulene, β-caryophyllene, patchoulol, and farnesol. Meanwhile, diterpenes, sesterterpenes, triterpenes, sesquarterpenes, and tetraterpenes are rarely detected in EOs due to their low volatility [39,40]. Figure 1 shows the chemical structures of common EO constituents.

### 2.2. Cytotoxic EOs against Breast Cancer

Previous studies have reported the cytotoxic nature of EOs against breast cancer cells through different mechanisms of action. EOs trigger the death of cancer cells via apoptosis, necrosis, or cell cycle arrest. This involves the loss of mitochondrial potential, changes in pH gradient, an increase in the cell membrane fluidity, and a decrease in adenosine triphosphate (ATP) synthesis [41]. In addition, the toxicity potential of EOs is suggested to be governed by the type of organism. In eukaryotes, toxicity decreases as the lipophilic components of EOs increase. In prokaryotes, toxicity increases as the lipophilic components of EOs increase [4]. Owing to their promising cytotoxic property, some common EOs were extensively investigated for their in vitro cytotoxic effects on breast cancer cell lines (Table 1). In addition, studies have shown that certain EOs in combination with a chemotherapeutic drug enhanced the cytotoxic activity on cancer cell lines. Thus, only a small dose of a drug is needed when combined with EOs while maintaining the same cytotoxic effect [42]. Common EOs, originating from cinnamon, rose, thyme, chamomile, lavender, jasmine, lemon, agarwood, lemongrass, and citronella, were investigated for their cytotoxic effects against human breast cancer MCF-7 cells. MCF-7 cells are often used in breast cancer studies owing to their ideal characteristics of the mammary epithelium. Physiologically, MCF-7 cells are hormone-responsive breast cancer cells, which express oestrogen receptors (ERs). ERs are nuclear proteins regulating the expression of specific genes in breast cancer development and progression, and approximately 80% of breast cancers are ER-positive. Thus, the MCF-7 cell line is suitable to be used as an experimental model for drug discovery in breast cancer [8].

In a study conducted by Zu and co-workers, thyme (*Thymus vulgaris*) EOs showed the most potent cytotoxic effect against MCF-7 cells with an inhibitory concentration of 0.030% (*v*/*v*). Other EOs from cinnamon, rose, chamomile, lavender, jasmine, and lemon showed cytotoxic effects ranging from 0.072 to 0.143% (*v*/*v*). The exhibited cytotoxic activity could be attributed to the different constituents present in the EOs, notably terpenes [43]. Lemongrass (*Cymbopogon citratus*) and citronella grass (*Cymbopogon nardus*) are two closely related medicinal herbs native to tropical countries. These aromatic plants have been investigated for their potential against cancer cells due to their excellent pharmacological properties. Lemongrass EOs exhibited a significant inhibitory concentration (IC_50_) at 0.28% (*v*/*v*), while citronella grass EOs showed an IC_50_ at 0.46% (*v*/*v*). Previous studies have demonstrated that the amount of major constituents in EOs plays a vital role in biological activities. Citronellal, a major constituent in citronella grass EOs, has been evaluated for its selectivity index (SI) against cancerous MCF-7 cells and non-cancerous cells. The SI in cytotoxicity is a measure used to assess the relative toxicity of a substance to different cell types, typically cancer cells versus non-cancerous cells. A higher SI suggests that a substance is more toxic to cancer cells, indicating potential therapeutic value with lower toxicity to non-cancerous cells [8]. Citronellal showed a high SI of 25.8, indicating its high selectivity towards cancerous MCF-7 cells [44,45]. In addition, agarwood (*Aquilaria* spp.) EOs have been assessed for their cytotoxic potential against MCF-7 cells. It was reported that 44 μg/mL of agarwood EOs could kill 50% of MCF-7 cells. Traditionally, agarwood is mainly used for religious, aromatherapy, and medicinal purposes. The observed cytotoxic effects might be due to the predominance of sesquiterpenoids, namely isoaromadendrene epoxide, agarospirol, and β-guaiene, in the agarwood EOs [46,47]. The ginger plant is popularly used in culinary and folk medicine. Ginger EOs extracted using hydrodistillation have been assessed for cytotoxicity against MCF-7 cells using an MTT assay. The results revealed the cytotoxic potential of ginger EOs against MCF-7 cells with an IC_50_ of 82.6 ± 3.2 μg/mL [48]. Tea tree, scientifically known as *Melaleuca alternifolia*, is highly regarded for its folk uses. A steam-distilled tea tree EO was evaluated for its cytotoxic potential against MCF-7 cells. A terpinen-4-ol-rich tea tree EO showed an IC_50_ of 537 μg/mL after 24 h treatment against MCF-7 cells. In addition, the findings revealed that tea tree EOs induced early-stage apoptosis against MCF-7 cells at concentrations of 100 and 300 μg/mL [49]. *Citrus limon* is a widely known fruit tree from the Rutaceae family. The leaf and branch of *C. limon* were hydrodistilled to give 0.01% (*v*/*w*) and 0.005% (*v*/*w*) oil yield, respectively. Based on the results, leaf EOs of *C. limon* demonstrated an IC_50_ of 10% (*v*/*v*) against MCF-7 cells. In addition, the leaf EOs induced apoptosis through increased expression levels of caspase-8 [50]. In another study, a monoterpenoid-rich lavender EO was studied for its cytotoxic potential against human breast MDA-MB-231 cancer cells. The lavender EO showed a dose-dependent cytotoxic effect on MDA-MB-231 cells with an IC_50_ of 0.259 ± 0.089 μg/mL. It was reported that a high content of eucalyptol is positively correlated with the observed cytotoxicity [51]. Basil is a widely used culinary herb worldwide. In the study conducted by Aburjai in 2020, basil EOs were dominated by linalool (36.26%) and eucalyptol (11.36%). In the MTT assay, basil EOs exhibited an IC_50_ of 432.3 ± 32.2 and 320.4 ± 23.2 μg/mL on MDA-MB-231 and MCF7 cells, respectively. It was hypothesised that the major constituents present in the basil EOs play a main role in the observed cytotoxic effects [52]. In a study by Niksic et al. (2021), thyme EOs were reported to inhibit cancer cell proliferation in a dose-dependent manner. A thymol and *p*-cymene-rich thyme EO was reported to exhibit an LC_50_ value of 60.38 μg/mL in a brine shrimp lethality assay. Meanwhile, the IC_50_ against MCF-7 cancer cells was recorded at 52.65 μg/mL [42]. *Matricaria recutita* (chamomile) is a medicinal herb commonly used in aromatherapy and folk medicine. Chamomile EOs were predominated by terpenoids which accounted for 63.51% of the total oil. In a cytotoxicity assessment, chamomile EOs showed potent cytotoxicity on MDA-MB-231 cells with an IC_50_ of 4 μg/mL. In addition, chamomile EOs reduced the migration and invasion of MDA-MB-231 cells. Based on these findings, it was suggested that the cytotoxic effects of chamomile EOs were due to the inhibition of PI3K/Akt/mTOR signalling pathway in MDA-MB-231 cells [53]. Overall, the demonstrated biological activity might be primarily attributed to the volatile constituents present in the EOs. Nonetheless, the chemical complexity of EOs plays a key role as each constituent contributes to the exhibited bioactivity and may regulate the biological effects of other constituents [54,55].

**Table 1:** Cytotoxic effects of some common EOs against breast cancer cells.

Breast Cancer Cells	EO	Botanical Source	IC_50_	Reference
MCF-7	thyme	*Thymus vulgaris*	0.030% (*v*/*v*)	[43]
	chamomile	*Anthemis nobilis*	0.072% (*v*/*v*)	
	rose	*Rosa centifolia*	0.074% (*v*/*v*)	
	cinnamon	*Cinnamomum zeylanicum*	0.076% (*v*/*v*)	
	jasmine	*Jasminum grandiflora*	0.077% (*v*/*v*)	
	lavender	*Lavandula stoechas*	0.142% (*v*/*v*)	
	lemon	*Citrus limonum*	0.143% (*v*/*v*)	
MCF-7	lemongrass	*Cymbopogon citratus*	0.28% (*v*/*v*)	[44]
	citronella grass	*Cymbopogon nardus*	0.46% (*v*/*v*)	
MCF-7	agarwood	*Aquilaria* spp.	44 μg/mL	[46]
MCF-7	ginger	*Zingiber officinale*	82.6 ± 3.2 μg/mL	[48]
MCF-7	tea tree	*Melaleuca alternifolia*	537 μg/mL	[49]
MCF-7	lemon	*Citrus limon*	10% (*v*/*v*)	[50]
MDA-MB-231	lavender	*Lavandula stoechas*	0.259 ± 0.089 μg/mL	[51]
MCF-7	basil	*Ocimum basilicum*	320.4 ± 23.2 µg/mL	[52]
MDA-MB-231			432.3 ± 32.2 µg/mL	
MCF-7	thyme	*Thymus vulgaris*	52.65 μg/mL	[42]
MDA-MB-231	chamomile	*Matricaria recutita*	1.5 μg/mL	[53]

### 2.3. Limitations of EOs

Even though EOs possess a wide range of bioactivity, their use is limited by their volatility, hydrophobicity, and instability. EOs comprise about 95% volatile and 5% non-volatile constituents [56]. Occasionally, the volatile constituents are affected by many external variables. The quality of EOs may be impacted by environmental variables such as air, heat, and irradiation. In addition, EOs’ hydrophobicity has led to their insolubility in a water-based medium. Plant EOs are unstable due to their thermolability. They can readily oxidise or hydrolyse during processing and storage. When exposed to environmental stimuli, they are vulnerable to oxidation, chemical changes, or polymerisation [57]. For example, EOs obtained from citrus trees contain a high concentration of monoterpenes, particularly dextrolimonene. However, they oxidise on contact with air. When dextrolimonene reacts with oxygen in the air, a new constituent is produced, which poses adverse effects [58]. Park and co-workers conducted research on EOs from *Kunzea ambigua*. The EOs were stored under various settings to examine the changes in colour and chemical compositions. A significant colour variation was observed when the EOs of *K. ambigua* were stored at room temperature under a light. On the contrary, the colour of EOs stored at freezing temperature, under refrigeration, or under room temperature without light was more stable. The chemical composition of EOs was generally constant throughout storage at room temperature, under refrigeration, and at freezing temperature in the absence of light. However, the amount of germacrene D, β-caryophyllene, and α-humulene was found to decrease significantly in the EOs of *K. ambigua* when exposed to light, possibly due to isomerisation [59].

## 3. Nanoencapsulation

**Figure 2:**
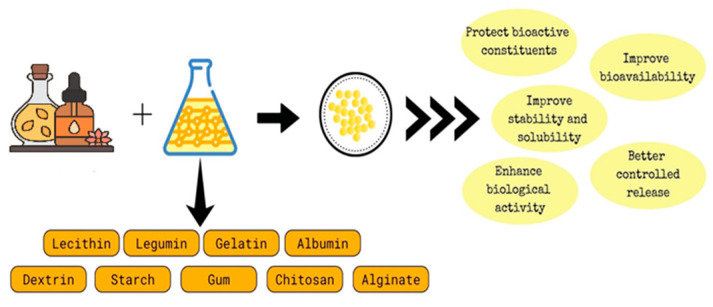
Nanoencapsulation of EOs with improved properties.

Nanoencapsulation is an emerging technique used to encapsulate bioactive constituents (the core material) inside secondary/wall materials (the matrix or shell) to produce nanocapsules [60]. It protects the bioactive constituents from light, heat, air, and moisture. Nanoencapsulation aids in minimising the evaporation of EOs and improving the delivery of bioactive constituents through controlled release. It converts liquid EOs into solid nanoencapsulated EOs and facilitates their applications [10]. The choice of an appropriate wall material is primordial in nanoencapsulation. Lecithin, legumin, gelatin, and albumin are among the proteins that are often employed as wall materials. They are naturally amphipathic, which makes them effective emulsifiers. Moreover, proteins are organic, environmentally friendly, and biodegradable. Bioactive components are also encapsulated in polysaccharides such as dextrin, starch, gums, CS, and alginates. Various wall materials have been used in research to encapsulate bioactive components to increase their bioactivity [61]. Figure 2 shows the nanoencapsulation of EOs with improved properties. For instance, it has been reported that the nanoencapsulation of eugenol with CS increased the antifungal and aflatoxin B1 inhibitory efficacy in stored rice [62]. Poly(lactide-co-glycolide) was used to nanoencapsulate bioactive *Trachyspermum ammi* seed EOs for possible use in the therapy of colon cancer. Based on the in vitro findings, the synthesised nanoencapsulated EO system triggered apoptosis by expressing apoptotic genes in human colon cancer cells [63].

### 3.1. Chitosan (CS)

CS is a naturally occurring polymer made up of *N*-acetyl-D-glucosamine and D-glucosamine units. It is the second most abundant polysaccharide in nature. In industrial settings, chitin undergoes deacetylation to form CS, wherein the acetamide group is converted into an amino group [64,65]. The resulting CS polymer may vary in length based on the conditions and parameters employed during the deacetylation process. CS typically exhibits molecular weights ranging from 300 to 1000 kDa, influenced by its degree of acetylation. Generally, CS is insoluble in water at neutral pH. However, the protonation of its free amino groups has made CS soluble in dilute acids. This pH-responsive solubility can be advantageous in drug delivery to specific regions of the body with varying pH levels. In addition, the number of amino groups present in CS may impact its mucoadhesion and transfection properties. This can enhance its residence time at the target site compared to other polymers, improving the overall efficiency of drug delivery [66]. Chitin, the precursor to CS, can be obtained from the exoskeletons of crustaceans and insects. CS is highly regarded as a safe and environmentally friendly material. It is non-toxic, biodegradable, and biocompatible. CS breaks down into non-toxic byproducts over time. This characteristic is advantageous in applications where the carrier needs to be gradually eliminated from the body. Owing to these distinctive attributes, CS is used in various applications such as pharmaceuticals, biomedicine, agriculture, and food [64,65]. The general properties of CS are listed in Table 2. 

In biomedical and pharmaceutical applications, the selection of the right molecular weight, degree of acetylation, and purity of CS are vital. According to the European Pharmacopoeia, the acceptance criteria for impurities present in CS are as follows: total impurities/insoluble ≤ 0.5%; heavy metals ≤ 40 ppm; sulphated ash ≤ 1.0%; no tolerance on iron and protein. In this aspect, the presence of proteins is critical as it may affect the biological effects of immunity. On the contrary, a high quantity of ash may influence the dissolution and the preparation of efficient CS-based drug delivery systems [66]. However, CS is sensitive to humidity due to its hygroscopic nature. The formation of hydrogen bonding promotes the retention of water on CS and affects its mechanical properties. In addition, CS is reported to thermally degrade at ambient temperature. Thus, low-temperature conditions are suggested for its storage [69]. CS is known to possess anticancer, antimicrobial, antidiabetic, antioxidant, and anti-inflammatory properties [64]. The encapsulation of EOs using CS has been reported to enhance the drug nature after successful drug delivery. In addition, CS has attracted a great deal of attention in drug co-delivery, gene delivery, and tissue engineering [65].

### 3.2. EO-Loaded CS Nanoparticles

**Figure 3:**
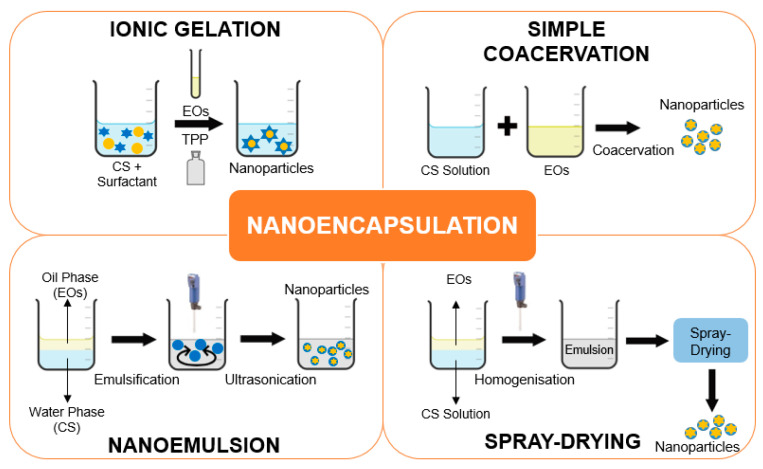
Various methods used to encapsulate EOs into CS nanoparticles.

Owing to the favourable attributes of CS, it is widely used in the nanoencapsulation of EOs. Various methods have been employed to encapsulate EOs into CS nanoparticles (Figure 3). Among them, ionic gelation is an economical method for encapsulating plant EOs. The method is eco-friendly and does not require high temperatures. Ionic bonding between the negatively charged cross-linking agents and the positively charged CS is involved in the synthesis of nanoparticles using ionic gelation [70]. In addition, a cross-linking agent such as sodium tripolyphosphate (TPP) is often used in the synthesis due to its biocompatibility and biodegradability attributes [71]. For instance, the ionic gelation method was used in the synthesis of *Cynometra cauliflora* EO-loaded CS nanoparticles. It involved the dissolution of CS in a diluted acetic acid solution containing a surfactant. Subsequently, EOs were added into the CS–surfactant solution followed by TPP. The synthesis was carried out under continuous stirring to facilitate precipitation. The synthesised *C. cauliflora* EO-loaded CS nanoparticles have shown potent cytotoxic effects against human breast cancer MCF-7 cells with an IC_50_ ranging from 3.72 to 17.81 μg/mL after a 72 h incubation period. Meanwhile, they showed cytotoxic effects against human breast cancer MDA-MB-231 cells with an IC_50_ ranging from 16.24 to 17.65 μg/mL after 72 h of treatment. The observed cytotoxicity was significantly enhanced as compared to that of the free EOs of *C. cauliflora* [8].

Another method used for encapsulating reactive and sensitive constituents in a nanoscale setting is coacervation. This method is separated into aqueous (hydrophilic constituents) and organic (lipophilic constituents) phases. In coacervation, however, organic solvents are required. A single polymer is used in simple coacervation. On the contrary, the formation of a matrix enclosing the bioactive constituents from two colloids with opposite charges is required in complex coacervation. Both proteins and polysaccharides are often used as oppositely charged polymers [70]. Compared to other nanoencapsulation methods, coacervation can reach a payload of over 99%. Coacervation regulates the release of bioactive constituents depending on temperature and mechanical stress [72]. In a study by Bastos and co-workers, black pepper EOs were encapsulated utilising the coacervation method. The study showed that the core material retained more than 80% of the terpenes present in the EOs [73].

Nanoemulsion involves a combination of two immiscible liquids. It can be achieved by combining an EO’s dispersion phase with water’s dispersed medium to create an oil-in-water emulsion [74]. The size of the synthesised EO nanoparticles can range from 10 to 1000 nm. This method serves as a delivery vehicle for targeted mechanisms of action because of the tiny size of the droplets produced in the nanoemulsion. The bioavailability of EOs is increased by nanometric-sized particles and a high surface-to-volume ratio [75]. In nanoemulsion, both high-energy and low-energy processes are appropriate. High-speed homogenisation or high-intensity ultrasonication is used to create high-energy nanoemulsions. This is essential in controlling the zeta potential, retention, and particle size of the synthesised nanoencapsulated system. According to the literature, oil-in-water nanoemulsion was used to encapsulate EOs from cinnamon, rosemary, and oregano. Among the encapsulated EOs, oregano EO nanoemulsions exhibited significant biological effects owing to their smaller droplet size, high encapsulation efficiency, and low separation phase [76].

Spray drying is a simple, inexpensive, and versatile encapsulation method. It is used to enhance the stability of EO constituents that are heat- and light-sensitive. Three main phases are typically involved in spray drying. In experiments, the emulsification of EO constituents occurs in a polymeric solution. Then, the emulsion is homogenised and atomised into a drying medium. A stable matrix is produced as a result of the high temperature used in this method. This method results in better nanoparticle quality and yield. In addition, the synthesised nanoparticles have high solubility and stability [70].

EO-loaded CS nanoparticles are superior in controlled release, stability, and solubility. In numerous investigations, CS nanoparticles were used in the delivery of bioactive EOs [13]. For example, CS nanoparticles have been successfully loaded with the EOs of lemongrass and clove. The synthesised nanoparticles had nanometric size and spherical shape. In comparison to the free EOs, the EO-loaded CS nanoparticles displayed enhanced biological activity [77,78,79]. In a study conducted by Hesami and co-workers, greater celandine EOs were encapsulated with CS. The encapsulation improved the cytotoxicity of the EOs against human breast cancer cells. The synthesised nanoparticles strongly induced apoptosis in the cancer cells. Hence, CS-based nanoparticles loaded with EOs are a vital source for chemoprevention and cancer suppression [80].

## 4. Cytotoxic Actions of EO-Loaded CS Nanoparticles against Breast Cancer Cells

CS-based nanoparticles are desirable for delivering bioactive EO constituents in targeted cancer therapy. To deliver the specific bioactive constituents to the target site, the administered particles must be tiny to pass through the microvasculature of cancer cells. Nanoparticles with appropriate particle size can enhance drug penetration and uptake via the cell membrane [81]. In general, EO-loaded CS nanoparticles are able to circumvent immune surveillance and attain target specificity. They penetrate the intracellular space of cancer cells and elude entrapment within endosomes to release bioactive EOs [82]. The effective delivery of bioactive EOs to the intracellular environment is crucial for attaining therapeutic purposes. Occasionally, physical techniques are employed to forcibly transport nanoparticles across the plasma membrane, and the uptake of nanoparticles primarily depends on the inherent endocytic uptake mechanism of the cancer cells [83].

It was reported that EO-loaded CS nanoparticles are able to circulate in the bloodstream for a relatively long time and accumulate at the cancer cell site [66]. This can be achieved through the enhanced permeability and retention (EPR) effect. In regard to this, the physicochemical properties (particle size, surface charge, and shape) of nanoparticles play a vital role in determining the EPR effect. They affect the amount and kinetics of nanoparticle accumulation at the target site. The penetration of EO-loaded CS nanoparticles into the vasculature of cancer cells was believed to occur via a passive targeting mechanism that effectively regulates pharmacokinetics and enhances overall therapeutic efficacy [82]. In addition, EO-loaded CS nanoparticles may deliver the bioactive EO constituents in a site-specific manner while reducing toxicity [66]. The demonstrated cytotoxic effects on cancer cells depend on the type and concentration of EO constituents. They are often regarded as not genotoxic [84]. According to a recent study, bioactive EOs damaged the inner cell membranes and organelles in eukaryotic cells by acting as prooxidants [85]. It was hypothesised that EO constituents reduce the size of cancer cells. They induced the death of cancer cells via apoptosis, necrosis, cell cycle arrest, and disruption of cell organelles [41].

There are several processes that control the release of EOs from CS nanoparticles. These include polymer swelling, diffusion through the polymeric matrix, and erosion or degradation of the polymer. It was postulated that EO-loaded CS nanoparticles release the first burst of bioactive constituents as a result of the polymer swelling, the formation of pores, or the diffusion of the bioactive constituents from the polymer’s surface [69]. In a study by Shetta and co-workers, a two-stage release pattern from CS nanoparticles was observed. Peppermint and green tea EOs were encapsulated into CS nanoparticles with an average size of 20 to 60 nm. Under different pH levels, the in vitro results showed a two-stage release pattern from phosphate-buffered saline (PBS) and acetate buffers. The initial burst was noticed for up to 12 h, while the gradual release remained for 72 h. Additionally, the findings showed that the acetate buffer had a higher release rate than the PBS, with full release of 75%. The release kinetics for both buffers was studied and found to follow the Fickian model [86]. 

Various studies have been conducted to study the cytotoxic effects of EOs loaded with CS nanoparticles against breast cancer cells (Table 3). According to Onyebuchi and Kavaz, an *Ocimum gratissimum* EO encapsulated in CS nanoparticles decreased the viability of MDA-MB-231 cells to 37.44% as compared to the unencapsulated EOs. Blebbing of membrane in breast cancer cells was observed upon treatment with *O. gratissimum* EO-loaded CS nanoparticles, suggesting the occurrence of apoptosis [87]. In a previous study, *Zataria multiflora* EOs were reported to possess antiproliferative properties against breast cancer cells. The EOs of *Z. multiflora* induced the breakdown and oxidation of DNA strands without affecting non-cancerous cells [88]. Encapsulation with CS significantly enhanced the cytotoxic activity against MCF-7 and MDA-MB-231 cells in a dose-dependent manner, with IC_50_ values of 21.2 and 6.2 µg/mL, respectively. In cell morphological study, nanoencapsulated-EO-treated MDA-MB-231 cells showed a pronounced loss in cell membrane structure and a noticeable nuclear fragmentation. Further, the sub-G1 population mass was triggered in a fluorescence-activated cell sorting analysis. In evaluating the MDA-MB-231 cell death mode, the *Z. multiflora* EO-loaded CS nanoparticles exhibited early apoptosis with noticeable chromatin condensation and cell shrinkage. This caused cell accumulation in the G2/M phase, while cells in the G1 phase increased nominally. It was reported that *Z. multiflora* EO-loaded CS nanoparticles triggered the production of intracellular reactive oxygen species (ROS) in the mitochondria, which leads to apoptosis. The observed apoptosis might be due to the oxidation and breakdown of the DNA strands. Based on the findings, an intercalative binding to the DNA helix is observed, suggesting the DNA-binding and -damaging properties of the *Z. multiflora* EO-loaded CS nanoparticles [89]. Traditionally, EOs of *Syzygium aromaticum* are used to treat wounds and burns. It was reported that the EOs possess antibacterial, anti-inflammatory, antioxidant, and anticancer properties. The nanoencapsulation of *S. aromaticum* EOs using CS (IC_50_: 45.89 µg/mL) exhibited significantly enhanced cytotoxic effects against MCF-7 cells as compared to those of its unencapsulated EOs (IC_50_: 172.47 µg/mL) [90]. In a study conducted by Valizadeh et al. (2021), CS nanoparticles containing *S. aromaticum* EOs were studied against human breast cancer MDA-MB-468 cells. The nanoencapsulated EOs showed improved cytotoxicity (IC_50_: 177 µg/mL) when compared to unencapsulated EOs (IC_50_: 243 µg/mL) [91]. EOs from *Citrus aurantium*, *Citrus limon*, and *Citrus sinensis* are predominated by limonene. CS nanoparticles containing *Citrus* EOs were prepared using an ionic gelation method. The synthesised CS-nanoencapsulated *Citrus* EOs exhibited improved cytotoxic properties against cancerous MDA-MB-468 cells. Among the *Citrus* EOs, a nanoencapsulated *C. sinensis* EO demonstrated the most significant cytotoxicity against MDA-MB-468 cells with an IC_50_ value of 23.65 µg/mL [92]. In addition, CS nanoparticles loaded with *Chelidonium majus* have been evaluated for chemotherapeutic potential against breast cancer. The recorded IC_50_ values of the nanoencapsulated leaf and root EOs of *C. majus* against MCF-7 cells were 41.5 and 77.6 µg/mL, respectively. Based on the findings, a nanoencapsulated leaf EO of *C. majus* triggered early apoptosis of MCF-7 cells [80]. The literature revealed that *Cinnamomum verum* EOs possess antiproliferative properties against MCF-7, HeLa, and Raji cells. In a study conducted by Khoshnevisan and colleagues, CS nanoparticles containing *C. verum* EOs were synthesised. They exhibited cytotoxic properties against MDA-MB-468 cells, with an IC_50_ value of 112.35 μg/mL [93]. Previous research has demonstrated the promising anticancer properties of *Curcuma longa* EOs. It was noticed that the unencapsulated EOs did not exert significant cytotoxicity towards MDA-MB-231 and MCF-7 cells. Interestingly, an enhancement in cytotoxicity was observed for nanoencapsulated CS-loaded *C. longa* EOs against MDA-MB-231 (IC_50_: 99.11 µg/mL) and MCF-7 (IC_50_: 82.88 µg/mL) cells. It was postulated that the presence of the CD44 receptor in the MDA-MB-231 and MCF-7 cells plays a key role in the enhanced cytotoxicity. The structural similarity of CS with the natural ligand of the CD44 receptor may facilitate the binding of the nanoencapsulated EOs and promote endocytosis [94]. In a study conducted by Samling and co-workers, the leaf, twig, and fruit EOs of *Cynometra cauliflora* were nanoencapsulated using CS via ionic gelation method. It is worth noting that all nanoencapsulated EOs showed a pronounced enhancement in cytotoxicity against MCF-7 and MDA-MB-231 cells. Among them, the twig EO-loaded CS nanoparticles exhibited the lowest IC_50_ (3.72 µg/mL) against MCF-7 cells after 72 h of treatment. Furthermore, all the nanoencapsulated EOs showed no cytotoxicity against human breast non-cancerous MCF-10A cells [8]. In a recent study, *Cinnamon cassia* EOs were nanoencapsulated to enhance their cytotoxic effects against breast cancer cells. The nanoencapsulated EOs showed better cytotoxicity (IC_50_: 25.24 µg/mL) than unencapsulated EOs (IC_50_: 32.25 µg/mL) against MDA-MB-231 cells. It was postulated that the observed cytotoxic effects might be due to the cell membrane breakdown. The nanoencapsulated EOs of *C. cassia* inhibited the migration and invasion of MDA-MB-231 cells in a dose-dependent manner. In the analysis of ROS, superoxide dismutase (SOD), and malondialdehyde (MDA), the nanoencapsulated EOs showed an increase in ROS and MDA and a decrease in SOD. The intracellular reaction of ROS and peroxidation of lipids might be altered, leading to cell apoptosis. The nanoencapsulated EOs of *C. cassia* lowered the mitochondrial membrane potential and showed an increase in the JC-1 monomer level. They upregulated the expression of caspase-3 and AIF proteins, suggesting the occurrence of cellular apoptosis. In an in vivo study, an inhibition of tumour growth in mice transplanted with breast cancer cells was observed when the nanoencapsulated EOs were administered at a dose of 25 mg/kg. The occurrence of cell apoptosis induced by nanoencapsulated EOs was further confirmed with TUNEL staining with the detection of a significant number of brown apoptotic cells. The nanoencapsulated EOs decreased the expression of the Ki-67 protein, indicating their potential in inhibiting the proliferation of transplanted tumour cells [95].

## 5. Conclusions

EOs, characterised by a variety of volatile constituents with untapped medicinal potential, offer promising prospects for health. Due to the limitations of EOs in specific biomedical applications, this study emphasises the importance of employing nanodelivery with the biopolymer CS. Advances in nanotechnology provide benefits such as enhanced stability, solubility, bioavailability, and controlled release compared to free EOs. Notably, CS-based nanoencapsulated EOs have gained significant attention, becoming a focal point in the development of an alternative drug release system. This approach improves the release profiles of bioactive EOs and enhances selectivity for targeted cells, thereby increasing the effectiveness of EOs against breast cancer cells when embedded in a polymeric matrix. The synergy between nanotechnology and bioactive EOs has the potential to expand their applications in the pharmaceutical and biomedical fields beyond traditional uses. The exploration of CS-based nanoencapsulated EOs signifies a promising pathway with implications for further translation into clinical applications in the treatment of breast cancer. 

## Figures and Tables

**Table 2:** General properties of CS.

Property	Description	Reference
Appearance	Semicrystalline of white or slightly yellow	[64]
Solubility	Soluble in diluted acid below pH 6.0. Insoluble in water and organic solvents	[67]
Molecular weight (Mw)	Low Mw: <100 kDa Medium Mw: 100 to 1000 kDa High Mw: >1000 kDa	[68]

**Table 3:** Cytotoxic effects of CS-based nanoencapsulated EOs against breast cancer cells.

Breast Cancer Cells	EOs	Plant Parts	Cell Viability/IC_50_	Reference
MDA-MB-231	*Ocimum gratissimum*	Leaf	Unencapsulated: 44.25%	[87]
			Nanoencapsulated: 37.44%	
MDA-MB-231	*Zataria multiflora*	Aerial	Unencapsulated: 30.5 µg/mL	[89]
			Nanoencapsulated: 6.2 µg/mL	
MCF-7			Unencapsulated: 33.1 µg/mL	
			Nanoencapsulated: 21.2 µg/mL	
MCF-7	*Syzygium aromaticum*	Bud	Unencapsulated: 172.47 µg/mL	[90]
			Nanoencapsulated: 45.89 µg/mL	
MDA-MB-468	*Syzygium aromaticum*	-	Unencapsulated: 243 µg/mL	[91]
			Nanoencapsulated: 177 µg/mL	
MDA-MB-468	*Citrus aurantium*	-	Unencapsulated: 2037.53 µg/mL	[92]
			Nanoencapsulated: 240.44 µg/mL	
	*Citrus limon*	-	Unencapsulated: 137.03 µg/mL	
			Nanoencapsulated: 40.32 µg/mL	
	*Citrus* *sinensis*	-	Unencapsulated: 168.00 µg/mL	
			Nanoencapsulated: 23.65 µg/mL	
MCF-7	*Chelidonium majus* L.	Leaf	Unencapsulated: 90.2 µg/mL	[80]
			Nanoencapsulated: 41.5 µg/mL	
		Root	Unencapsulated: 126.4 µg/mL	
			Nanoencapsulated: 77.6 µg/mL	
MDA-MB-468	*Cinnamomum verum*	-	Unencapsulated: -	[93]
			Nanoencapsulated: 112.35 µg/mL	
MDA-MB-231	*Curcuma longa*	-	Unencapsulated: 329.53 µg/mL	[94]
			Nanoencapsulated: 99.11 µg/mL	
MCF-7			Unencapsulated: 344.60 µg/mL	
			Nanoencapsulated: 82.88 µg/mL	
MCF-7	*Cynometra cauliflora*	Twig	Unencapsulated: NS	[8]
			Nanoencapsulated: 7.69 µg/mL	
		Fruit	Unencapsulated: NS	
			Nanoencapsulated: 17.81 µg/mL	
		Leaf	Unencapsulated: NS	
			Nanoencapsulated: 3.72 µg/mL	
MDA-MB-231		Twig	Unencapsulated: NS	
			Nanoencapsulated: 16.24 µg/mL	
		Fruit	Unencapsulated: NS	
			Nanoencapsulated: 17.65 µg/mL	
		Leaf	Unencapsulated: NS	
			Nanoencapsulated: 16.48 µg/mL	
MDA-MB-231	*Cinnamon cassia*	-	Unencapsulated: 32.25 µg/mL	[95]
			Nanoencapsulated: 25.24 µg/mL	

Note: NS = not significant.

## Data Availability

Not applicable.
